# From "Weight of Evidence" to Quantitative Data Integration using Multicriteria Decision Analysis and Bayesian Methods

**DOI:** 10.14573/altex.1412231

**Published:** 2015

**Authors:** Igor Linkov, Olivia Massey, Jeff Keisler, Ivan Rusyn, Thomas Hartung

**Affiliations:** 1 US Army Engineer Research and Development Center, Concord, MA, USA; 2 MIT, Cambridge, MA, USA; 3 University of Massachusetts, Boston, MA, USA; 4 Texas A&M University, College Station, TX, USA; 5 Johns Hopkins Bloomberg School of Public Health, Center for Alternatives to Animal Testing, Baltimore, MD, USA; 6 University of Konstanz, Center for Alternatives to Animal Testing Europe, Konstanz, Germany

**Keywords:** weight of evidence, decision analysis, Bayesian, toxicology, policy

## Abstract

“Weighing” available evidence in the process of decision-making is unavoidable, yet it is one step that routinely raises suspicions: what evidence should be used, how much does it weigh, and whose thumb may be tipping the scales? This commentary aims to evaluate the current state and future roles of various types of evidence for hazard assessment as it applies to environmental health. In its recent evaluation of the US Environmental Protection Agency’s Integrated Risk Information System assessment process, the National Research Council committee singled out the term “weight of evidence” (WoE) for critique, deeming the process too vague and detractive to the practice of evaluating human health risks of chemicals. Moving the methodology away from qualitative, vague and controversial methods towards generalizable, quantitative and transparent methods for appropriately managing diverse lines of evidence is paramount for both regulatory and public acceptance of the hazard assessments. The choice of terminology notwithstanding, a number of recent Bayesian WoE-based methods, the emergence of multi criteria decision analysis for WoE applications, as well as the general principles behind the foundational concepts of WoE, show promise in how to move forward and regain trust in the data integration step of the assessments. We offer our thoughts on the current state of WoE as a whole and while we acknowledge that many WoE applications have been largely qualitative and subjective in nature, we see this as an opportunity to turn WoE towards a quantitative direction that includes Bayesian and multi criteria decision analysis.

## 1 Introduction

The inexact science of converting existing environmental health and toxicology knowledge into risk management decisions and policy relies on a growing volume of increasingly diverse scientific data. Past work in this area was guided by a small number of experimental techniques and models. Today, however, toxicity data is much more diverse. It can be collected by different modes, compounded with experiments or models, and often point in different directions regarding the same assessment endpoint. Individual lines of evidence constitute an information base from which conclusions must be drawn regarding public health and economic development. Weight of evidence (WoE) is an approach that, by means of qualitative or quantitative methods, integrates individual lines of evidence to form a conclusion (Linkov et al., 2009) and has been widely used in both ecological and human health risk assessments to collate heterogeneous information and justify selection of regulatory benchmarks. Its use in the process of validation of new and alternative test methods has been explored earlier (Balls et al., 2006).

WoE, as an approach, is currently at a crossroads. While there are significant efforts to formalize WoE methodologies, voices of skepticism about the approach’s utility exist as well. For example, the Organisation for Economic Co-operation and Development (OECD) aims to formalize WoE as a process for developing adverse outcome pathways (AOPs) (OECD, 2014). On the other hand, the National Research Council (NRC) review of the EPA’s Integrated Risk Information System (IRIS) process concluded that WoE “has become too vague and is of little scientific use” (NRC, 2014a). In its place, the NRC proposes alternative methods of varying quantitative nature such as read-across (Patlewicz et al., 2014) and systematic review (Rooney et al., 2014; Hoffmann and Hartung, 2006). The question of whether WoE is a legitimate tool that should continue to be developed and formalized or an obsolete concept that should be disregarded and replaced by something else altogether is thus a subject of significant interest.

We argue that regardless of the name, integration of individual lines of evidence is an essential component of environmental assessments that should be standardized to establish consistency and comparability across similar efforts. Weed (2005) reviewed WoE applications in environmental health and Linkov et al. (2009) enhanced the review and proposed a WoE taxonomy to categorize analyses on the degree of quantitative rigor. We believe that criticism of WoE by the NRC and others is related to what these reviews call “colloquial WoE use” while approaches endorsed by OECD and other proponents refer to more advanced quantitative WoE tools. Moreover, we argue that the application of Bayesian tools that were in fact integral to the initial conception of WoE in the 1960s would enhance the information base and rigor of WoE applications. Finally, we propose multi criteria decision analysis (MCDA) as a proxy for more advanced Bayesian tools as a way for evidence integration under high uncertainty. We thus call for further standardization of WoE tools and provide historical and methodological perspectives to support this aim.

## 2 WoE emergence and method taxonomy

Even though WoE evaluation can be dated back to Greek mythology with images of Themis, the goddess of law and order, holding two scales presumed to hold the balance of weights for or against a certain hypothesis, it was Professor I. J. Good of Virginia Polytechnic Institute and State University who first proposed the WoE methodology as an inherently Bayesian statistical approach (Good, 1960). A Bayesian model is based on updating “prior” beliefs for or against a particular hypothesis after evaluation of information or evidence in order to achieve a “posterior” belief. Bayes’ rule of 1763 is typically summarized as Pr(A|B) = Pr(B|A) Pr(A)/Pr(B), where Pr(A) and Pr(B|A) represent prior beliefs about the likelihood that A is true and that B is also true, on the condition that A holds. When new information is obtained that B holds, Bayes’ rule is used to calculate an updated probability that A holds, i.e., Pr(A|B).

Results of this rule can be expressed in various ways, notably with the Bayes factor, i.e., the ratio of the posterior odds to the prior odds. In this case, WoE is defined as the logarithm of the Bayes factor (Good, 1985). By the rules of logarithms, this ratio has an additive property, which is desirable for creating simple scoring rules to evaluate information (Good, 1984). Bayesian models, updating, Bayes factors and their relationship to WoE are explained in much more detail in Good (1960, 1984, 1985, 1989).

**Fig 1:**
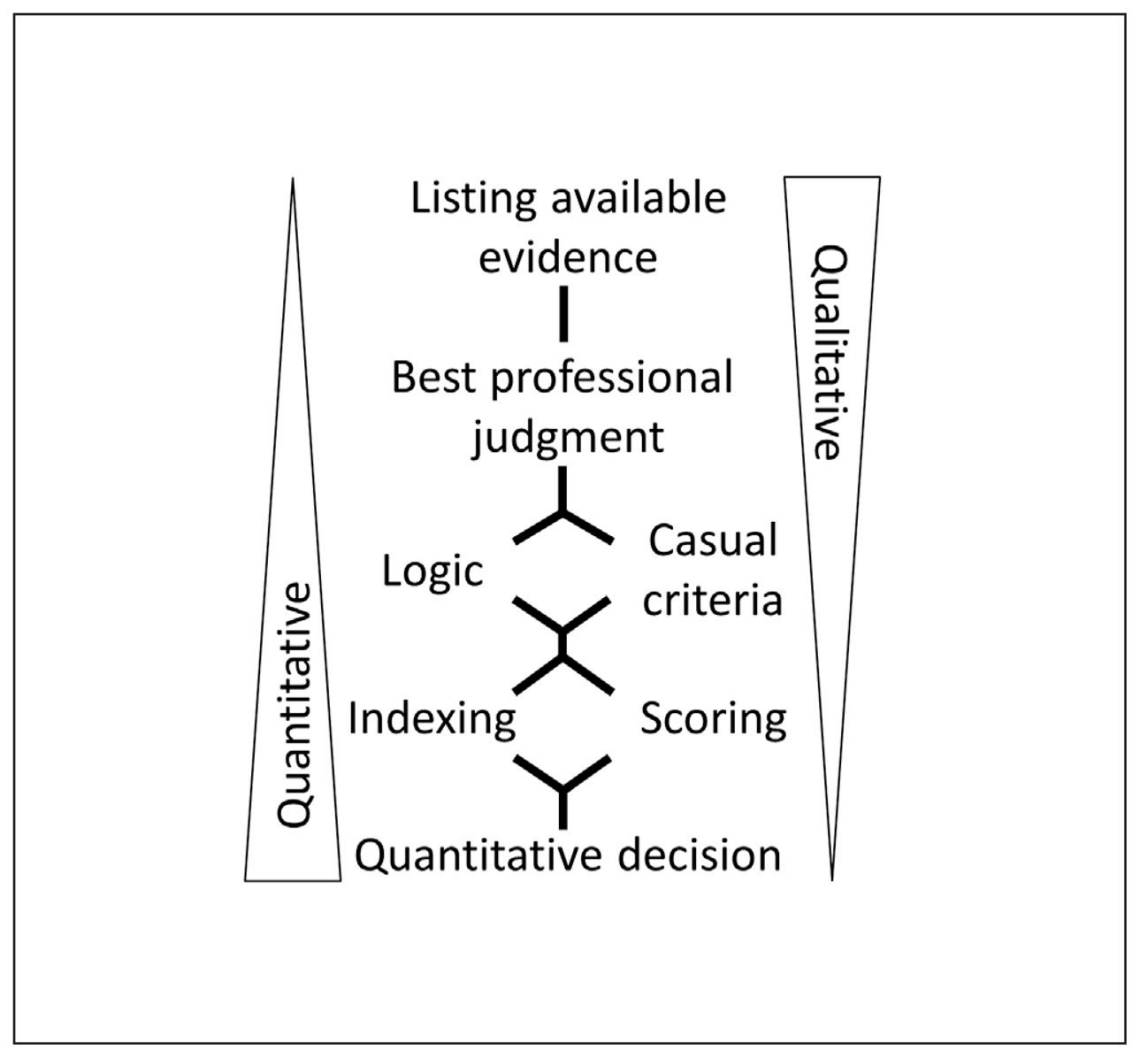
Classification of WOE approaches After Linkov et al., 2009 with permission.

While Good continued to expound on the mathematical relationships linking probability and Bayesian models to WoE into the 1980s, the US EPA and other regulatory agencies started to use the same terminology for very different analytical processes (see HERA, 2002 for review). When its applications are scrutinized and categorized, it becomes apparent that the WoE methodology, as it was conceived, has diverged and become diluted (Weed, 2005). Linkov et al. (2009) developed the structure in Figure 1 to classify different WoE approaches that have been applied in human health and ecological risk assessment. Methods are classified by the degree to which they are quantitative: the least quantitative and simplest methods are categorized as Listing Evidence, which consist of presenting evidence without any steps to integrate it, while the most quantitative and sophisticated, called Quantitative Methods, generally calculate risk probabilities using statistics (as the Bayesian Approach would) from various lines of evidence and use formal decision-analytical tools to assess hazard (Linkov et al., 2009).

## 3 Prevalent criticisms of WoE and recommendations

Practice of WoE has been scrutinized recently in several academic efforts as well as by National Academies. Weed (2005) characterized most WoE processes that were applied to human health-related assessments as largely qualitative and seldom rigorous in nature. In a review of 114 WoE articles on human health or ecological risk assessment published before 2000, it was similarly found that the vast majority of assessments employed the Best Professional Judgment method, considered to be largely qualitative (Linkov et al., 2009). As evidenced by its recent past applications, it thus seems that WoE has been increasingly used as a “colloquial” term to describe the consideration of qualitative evidence and has lacked structured guidelines.

Most recent criticism of WoE comes from the NRC review of EPA’s IRIS assessment of formaldehyde and methanol (NRC, 2014a). Broad aspects of general approaches and methods utilized by the EPA have also come under scrutiny. In the “Evidence Identification for Hazard Identification” section of the review, the NRC makes the startling determination that WoE is no longer of scientific use and recommends alternative quantitative and qualitative approaches for synthesizing evidence in order to answer pressing questions related to chemical hazard.

To replace WoE as a means for synthesizing multiple lines of evidence to determine hazard, NRC recommends a set of qualitative and quantitative approaches. The qualitative suggestions include Guided Expert Judgment and Structured Processes such as the Grading of Recommendations Assessment, Development, and Evaluation (GRADE) system. The NRC warns against utilizing the Bradford Hill (BH) criteria, which are often applied to analyze strength of association, consistency, specificity, temporality, biologic gradient, plausibility, coherence, experimental evidence and analogy (Hartung et al., 2013a,b), on the basis that “the Hill criteria cannot be taken as either necessary or sufficient conditions for an association to be raised to a causal association” (NRC, 2014a). It is ironic then that the committee goes on to recommend the GRADE system which is characterized as being “closely aligned with the Hill criteria for establishing causality” (NRC, 2014a). Guided Expert Judgment practices involve internal evidence integration by an individual or group of experts. This process, therefore, generally lacks transparency and reproducibility, thus making it impossible to create a systematic structure with clearly set forth guidelines for evidence integration. Given that WoE was criticized in the review for containing too much subjectivity, the suggestion to replace it with Guided Expert Judgment seems contradictive.

As for quantitative approaches, NRC recommends meta-analysis, probabilistic analysis, and the Bayesian approach as sufficient replacements for WoE to improve evidence integration in the IRIS process (NRC, 2014a). Meta-analysis and probabilistic bias analysis both provide quantitative estimates of an effect size by converting confidence intervals that account for uncertainty into quantitative judgment values. Meta-analysis is appropriate for combining data from similar studies through statistical methods, but its techniques are not well suited to accounting for biases.

Of the three quantitative evidence integration methods suggested as suitable replacements for WoE by NRC, the Bayesian approach is discussed most extensively. The Bayesian approach is noted as “an opportunity to include as much rigor in constructing a formal model of evidence integration and uncertainty as one wants… with a type of theoretical guarantee” (NRC, 2014a). Bayesian models are also praised for their ability to account for various types of uncertainty and integrate differing styles of evidence from studies (e.g., human, animal, mechanistic). A potentially prohibitive challenge to using a strictly Bayesian approach is that, in the face of data and cognitive limitations, detailed elicitation from experts must be conducted for proper modeling of probabilities and statistical relationships.

## 4 Effective alternatives and existing Bayesian approaches

Even though WoE approaches to hazard assessment have relied heavily on subjective inputs and qualitative analysis, Bayesian methods have been used as well. For example, the Optimized Strategies for Risk Assessment of Industrial Chemicals through Integration of Non-Test and Test Information (OSIRIS^1^) webtool offers a promising opportunity to validate quantitative WoE approaches using Bayesian statistics in a transparent and reproducible manner. Buist et al. (2013) and Rorije et al. (2013) both make use of the tool to implement Integrated Testing Strategies (ITS) (Hartung et al., 2013a). Buist et al. develop a WoE case to analyze mutagenicity that utilizes an independent Bayesian approach in the OSIRIS webtool, but note that data limitations were faced in the process which prevented evaluation of an entire set of quantitative-structure activity relationships (QSARs). Jaworska and Hoffmann (2010) advocate Bayesian approaches in ITS (Jaworska et al., 2010) later applied to skin sensitization as a test case (Jaworska et al., 2011). A similar case to assess skin sensitization is made by Rorije et al. in which Bayesians are applied to conclude sufficiency of a WoE approach in determining risk associated with chemicals. A formal mechanism for evidence integration using Bayesian belief networks (BBNs) and decision trees is presented in Small (2008) to assess uncertainty in models for cancer risk assessment. The study makes use of Netica BBN software for conducting Bayesian analysis, demonstrating the utility of existing software for future analyses that would benefit from Bayesian analysis.

While not directly related to toxicology, McClung (2011) and Sujatha et al. (2014) apply Bayesian statistics and probabilities to WoE approaches to assess risks associated with natural disasters such as landslides and avalanches. A Bayesian approach to WoE is additionally presented as a case study to estimate the probability of site impairment based on chemical, biological and toxicity data in the Great Lakes region in Smith et al. (2002).

Because the works mentioned above directly utilize a Bayesian approach to perform WoE, they are effectively integrating evidence in the style recommended by the NRC and Good. The fact that they incorporate WoE should not be a strike against these useful applications. Existing WoE approaches that are inherently quantitative and Bayesian should act as instructional guidelines for future applications of WoE.

## 5 Multi criteria decision analysis (MCDA) as a suitable proxy for Bayesian analysis

**Fig. 2:**
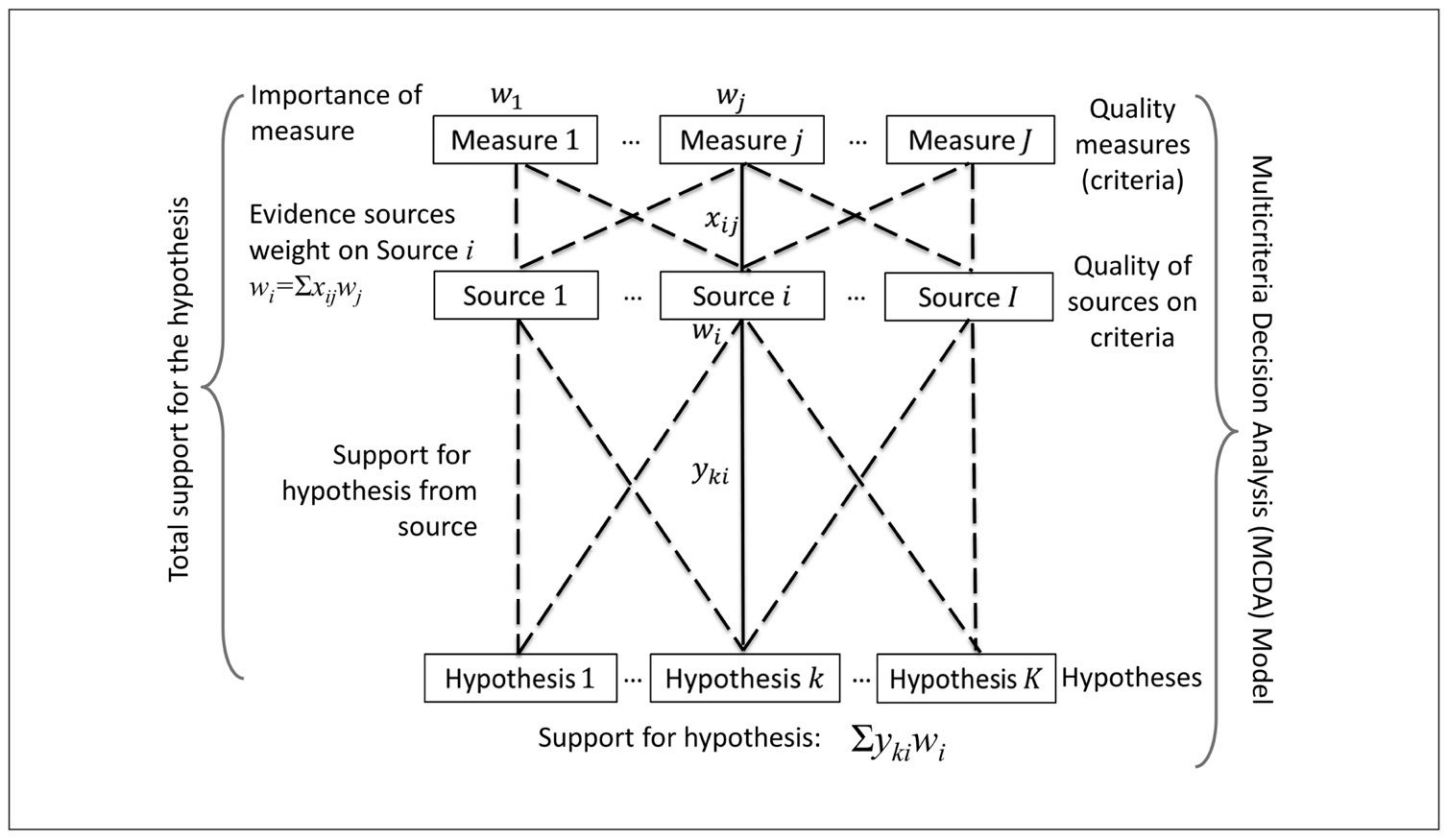
Use of MCDA for WOE evaluation

A systematic MCDA approach to WoE may serve, in many cases, as a suitable proxy for the Bayesian approach, such as when model formulation is restricted by data limitations. MCDA can facilitate the synthesis of multiple sources of evidence, e.g., linking *in vitro* and *in vivo* studies to assess hazard, as noted in another recent NRC report (NRC, 2014b). It is possible to combine different sources by considering how strong each is with respect to several criteria (Fig. 2). Thus, with evidence sources i = 1,I, we would define evidence quality measures j = 1, J. For a dependent variable of interest y, we denote the estimated value based on source i as y_i_. We denote the quality of information source i on criterion j as x_ij_. Finally, we would obtain judgments from experts using standard elicitation techniques such as swing weighting, i.e., about the relative importance of quality measures, w_j_. It is in this last piece that MCDA serves as a qualitative proxy for formal mathematics along the lines of Winkler and Clemen (2004), as well as Good’s original formulation, e.g., where a source is weighted proportionally to its precision (expertise), as well as its correlation with other sources. It may be possible in some cases to obtain detailed data and elicitations to derive Bayes factor weights for some of the most basic characteristics of data sources. But often this will not be practical or will not be sufficient to take full account of our understanding of data sources. Then other qualities of the evidence could be incorporated in the form of criteria within a multi-criteria model without requiring formal statistical modeling that is often not practical due to issues with reliability, specificity and relevance.

Using such a MCDA model, we can then calculate a relative weight for each evidence source i, w_i_ = Σ_j_ w_j_x_ij_. We can formulate a combined estimate of y = Σ_i_w_i_y_i_/Σ_i_w_i_. To use this in judging hypotheses, we might use k as an index for hypotheses and interpret the score y_k_ to be support for hypotheses k from the evidence, while y_ki_ is the support for the k^th^ hypotheses from the i^th^ source of evidence. In this way, the most favored hypothesis is the one with the highest score y_k_ = Σ_i_w_i_y_k i_/Σ_i_w_i_. Selecting a hypothesis amounts to applying a MCDA model with a two-level criteria hierarchy, where the first level is the sources of evidence, and the second level consists of the characteristics of those sources. In basic cases, this model should replicate a Bayesian approach, while in many other cases it should provide a good approximation to such an approach (although research is needed to understand just how robust MCDA approaches would be).

MCDA approaches to WoE have already been conducted in a quantitative manner. MCDA application to WoE assessment of nanomaterial hazard was recommended in Zuin et al. (2011) as an addition to a pre-existing WoE-based approach that consisted of expert judgment to rank hazards. A full conceptual framework for assessing nanomaterial hazard on material properties, toxicity and data quality criteria was developed thereafter (Hristozov et al., 2014). Linkov et al. (2011) apply MCDA to answer the common environmental health question of assessing risk for sediment contamination by using the sediment quality triad (SQT) as criteria for assessment.

## 6 Conclusion

As high impact regulatory decisions continue to be made in hazard assessment and other environmental health applications, the necessity to provide a clear basis for decision-making is becoming increasingly important. WoE, conducted in a manner consistent with its original intent, should be considered as having valuable scientific merit for evidence integration in toxicology. The present state of WoE, and evidence integration in general, is such that approaches to hazard assessments in toxicology differ greatly between applications, among both qualitative and quantitative assessments.

With such wide applicability of WoE approaches for decision- making, it is relatively simple to understand how general guidelines for application could have become convoluted over the years. Even Good himself noted a miscommunication in the exact definition of WoE during early correspondence with another mathematician (Good, 1960). The need to develop transparent, systematic guidelines for evidence integration in the toxicology community is not a finding that is unique to the NRC. There is a common assertion about the fundamental necessity of validating test methods as well as improving overall flexibility, transparency, consistency, reproducibility and objectivity of WoE across the literature. Hartung (2009) determined that methods for evidence integration are “urgently needed in toxicology” and, while it was noted how a “well-defined approach to weight-of-evidence in the field of Bayesian statistics” was laid out in Good (1985), it was also noted that there were data limitations for the approach to be used in toxicology at that time. The Evidence-based Toxicology Collaboration^2^ is promoting this concept (Stephens et al., 2013; Hoffmann et al., 2014). Increasing transparency and objectivity in literature-based environmental health assessments is also a primary focus in Rooney et al. (2014), but the evaluation model set forth in the study is largely qualitative in nature. Despite general agreement on the principles of evidence integration, regulatory acceptance of methods is limited by disagreement over their application to setting-specific cases (Hess et al., 2014).

In general, we expect the qualitative approaches recommended as alternatives to a WoE approach to become antiquated and to be of limited use for decision-making as scientific developments continue. Qualitative approaches, as they apply to WoE, will likely lack the transparency and objectivity to be accepted by regulatory science communities. This lack of clarity and systematic methodology similarly reflects the findings mentioned as justification for discarding WoE studies and declaring them to be of “little scientific use” (NRC, 2014a).

While we concede that some applications of WoE in the scientific community have been conducted in a subjective and fuzzy nature, WoE as a concept should not be completely discounted. WoE in the traditional Bayesian or MCDA style offers an opportunity to redirect evidence integration in toxicological assessments onto a more quantitative, transparent and objective path. In addition to their use in quantitative evaluation of individual lines, MCDA applications to WoE add the visual effect of a mapped decision structure (e.g., a hierarchical model), and more generally offer a transparent way to incorporate judgments even when a formal Bayesian approach cannot be used. Combined with more recent proposals to visualize the relative contributions of various data types through the Toxicological Priority Index (ToxPi) (Reif et al., 2013), MCDA or other data-integration tools may yield not only transparent and quantitative, but also easily communicable means for presenting complex evidence. Future applications of WoE should incorporate Bayesian statistics in a reproducible approach that is transparent, objective and quantitative. For those assessments that face data limitation for statistical probability evaluations, a rigorous and numerical MCDA approach to WoE can be an appropriate alternative to a Bayesian approach.

## Footnotes

1 http://www.ufz.de/osiris/index.php?en=15009

2 http://www.ebtox.com
